# Giant circumflex artery with bilobed saccular aneurysm and right atrial fistula mimicking a bi-atrial hydatid cyst: a case report

**DOI:** 10.1093/jscr/rjad108

**Published:** 2023-03-09

**Authors:** Aida Soufiani, Hamza Chraibi, Samah El-Mhadi, Zineb Agoumy, Zineb Fassi Fehri, Rokya Fellat, Adil Bensouda, Nesma Bendagha, Said Moughil

**Affiliations:** Cardiology A Department, Ibn Sina Hospital, Mohammed V University, Rabat, Morocco; Cardiology A Department, Ibn Sina Hospital, Mohammed V University, Rabat, Morocco; Cardiology A Department, Ibn Sina Hospital, Mohammed V University, Rabat, Morocco; Cardiology A Department, Ibn Sina Hospital, Mohammed V University, Rabat, Morocco; Cardiology A Department, Ibn Sina Hospital, Mohammed V University, Rabat, Morocco; Cardiology A Department, Ibn Sina Hospital, Mohammed V University, Rabat, Morocco; Cardiovascular Surgery B Department, Ibn Sina Hospital, Mohammed V University, Rabat, Morocco; Cardiology A Department, Ibn Sina Hospital, Mohammed V University, Rabat, Morocco; Cardiovascular Surgery B Department, Ibn Sina Hospital, Mohammed V University, Rabat, Morocco

**Keywords:** Coronary artery fistula, Coronary cameral fistula, Coronary aneurysm, Multimodal imaging

## Abstract

Coronary artery fistulas (CAF) are rare anomalies that pose a significant diagnostic and therapeutic challenge. Most of them originate from the right coronary artery and are congenital. They are often associated with coronary aneurysms. We report the case of a 38-year-old Black man who presented with exertion dyspnea. Transthoracic echocardiography found what was thought to be a bi-atrial hydatid cyst, alongside a right atrial shunt. Cardiac magnetic resonance imaging showed a cystic lesion hypointense on T1 and T2 sequences, located next to the left atrium as well as an aneurysmal circumflex artery shunting in the right atrium. Coronary angiography and computed tomography angiography confirmed the bilobed circumflex saccular aneurysm and CAF. The patient underwent a successful surgery, which consisted of closure of the fistula using two patches. He was discharged after an uneventful postoperative course. Our case report illustrates the diagnostic difficulty of CAF and the importance of multimodal imaging.

## INTRODUCTION

Coronary artery fistulas (CAF) are abnormal connections between coronary arteries and cardiac chambers (coronary cameral fistulas (CCF)) or major thoracic vessels. They are associated in 14% of cases with coronary aneurysms. The majority of CAF are congenital, and most patients are asymptomatic. Sometimes, they present symptoms of myocardial ischemia or left-to-right shunts [1].

In this paper, we report the case of 38-year-old man presenting with exertion dyspnea, in whom cardiac hydatic cyst was first suspected on initial evaluation, but more advanced imaging modalities allowed us the rectify the diagnosis.

## CASE REPORT

A 38-year-old Black man with no medical history or cardiovascular risk factors complained of Class II exertion dyspnea. He was admitted in the cardiology department for further exploration.

**Figure 1:**
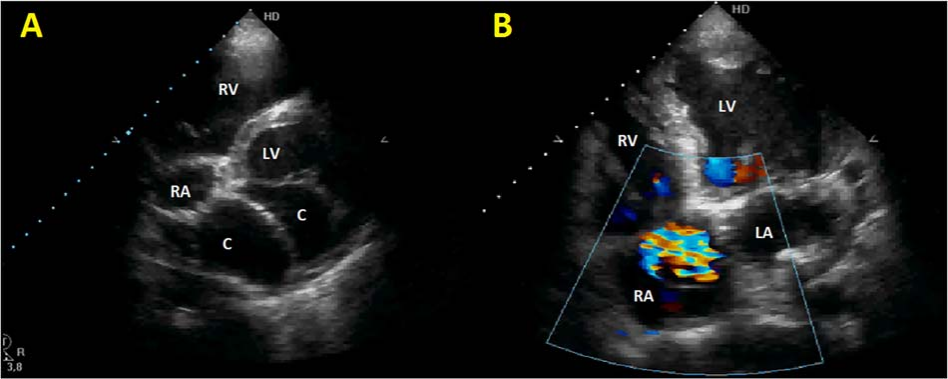
Initial TTE. (**A**) Parasternal short-axis view showing a bi-atrial cystic lesion; (**B**) apical 4-chamber view showing color Doppler aliasing in the RA, indicating a shunt. RV, right ventricle; LV, left ventricle; C, cystic lesion.

**Figure 2:**
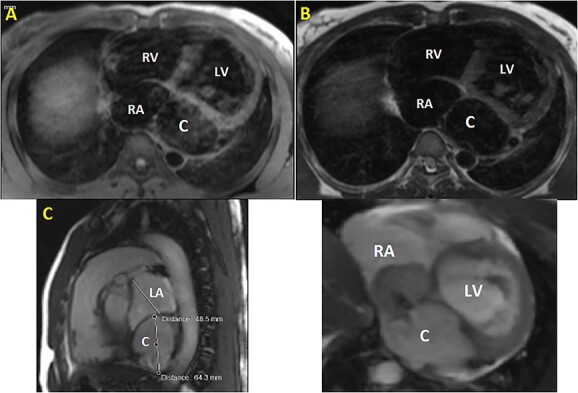
CMR. (**A**) T1 sequence showing a cystic hypointense lesion, with the same signal than cardiac chambers, located below and behind the LA; (**B**) T2 sequence showing the hypointense lesion; (**C**) vertical long axis view showing the bilobed lesion; (**D**) cine imaging showing an aneurysmal circumflex artery with a right atrial shunt. RV, right ventricle; LV, left ventricle; C, cystic lesion.

**Figure 3:**
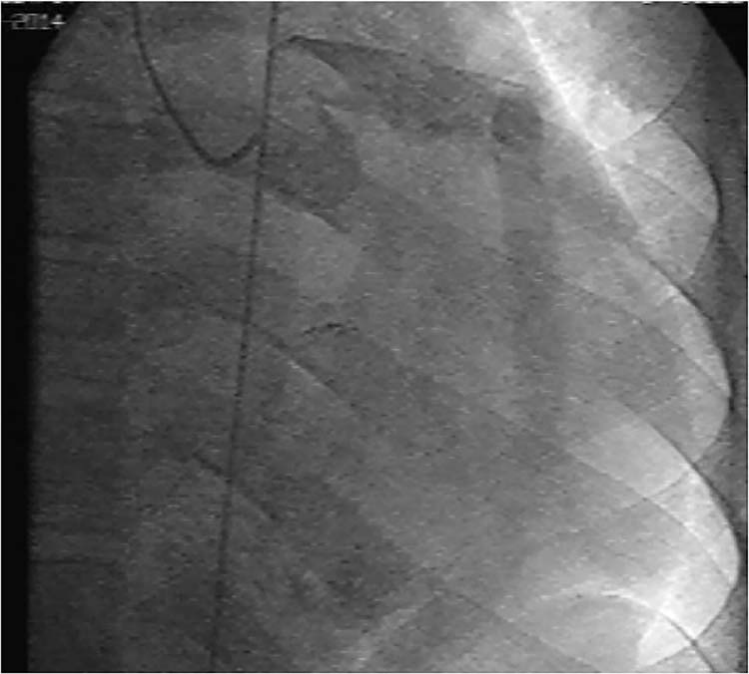
CA showing a giant aneurysmal circumflex artery.

**Figure 4:**
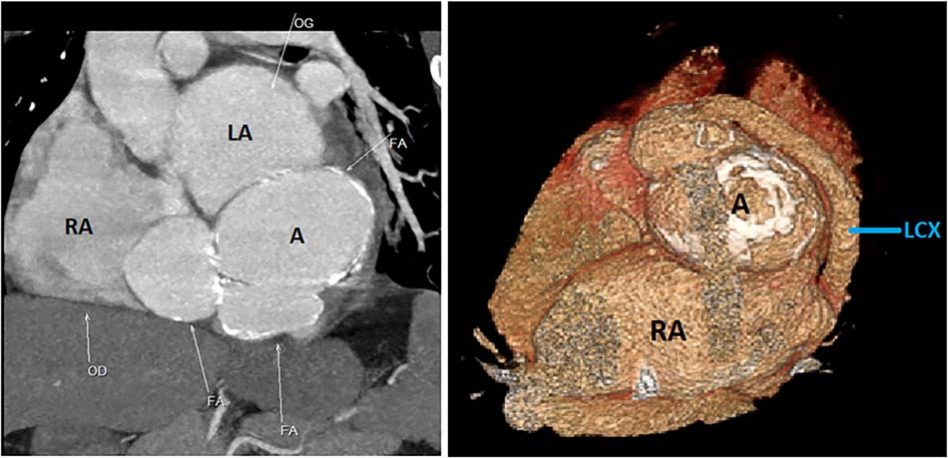
CCTA (with 3D reconstruction) showing the CAF alongside a bilobed saccular aneurysm fed by a giant circumflex artery, next to the RA. A, aneurysm of the circumflex artery; LCX, circumflex artery.

Initial physical examination showed stable vital signs. Auscultation found systolic murmurs of mitral and tricuspid regurgitations. There were no peripheral edema or other signs of heart failure. Abdominal examination revealed no tenderness, hepatomegaly or ascites. Electrocardiogram at admission showed sinus rhythm with no conduction abnormalities or signs of ischemia. Chest radiograph found an enlargement of the cardiac silhouette. Routine blood tests were normal. Transthoracic echocardiography (TTE) found a bi-atrial cystic structure, which we initially suspected to be a hydatid cyst as cystic echinococcosis is highly endemic in Morocco. Color Doppler showed a massive shunt with blood coming into the right atrium (RA) from an unknown source (Fig. 1). Cardiac magnetic resonance imaging (CMR) showed a bilobed cystic lesion hypointense on T1 and T2 sequences, with the same signal as cardiac chambers, located below and behind the left atrium (LA). The superior lobe was 48-mm wide and the inferior lobe 65-mm wide. On the peripheral wall of this structure, a giant circumflex artery (LCX), with a right atrial shunt, was present (Fig. 2). Coronary angiography (CA) along with computed tomography angiography (CCTA) confirmed the bilobed circumflex saccular aneurysm and CAF (Figs 3 and 4).

**Figure 5:**
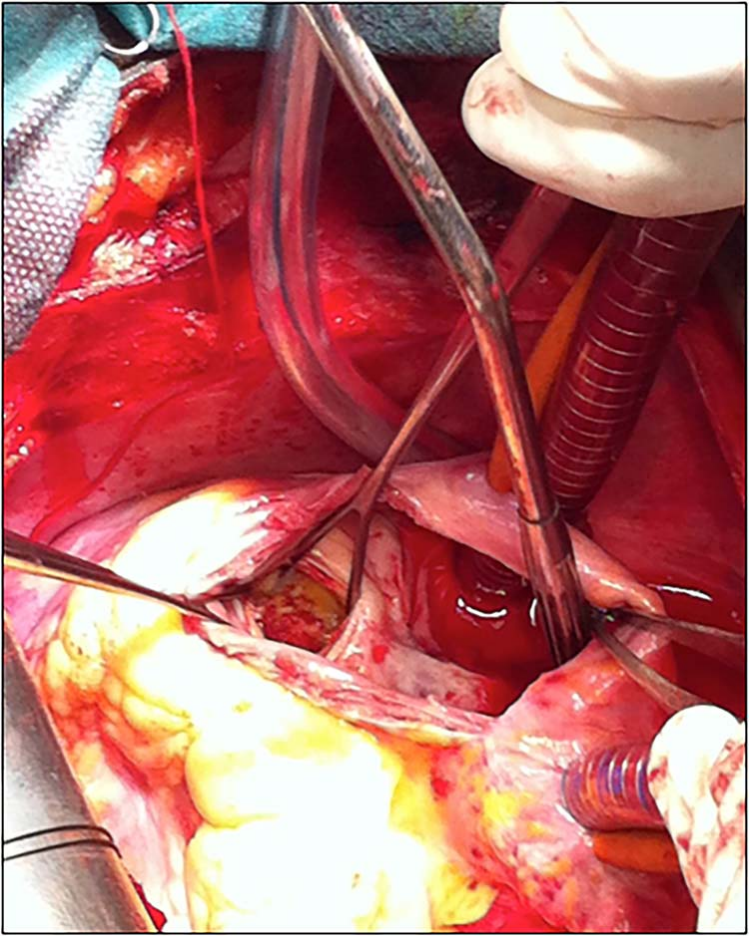
Picture of the surgery showing the patching of the CAF.

The patient underwent cardiac surgery with a median sternotomy approach and cardiopulmonary bypass. Exploration confirmed the presence of a giant aneurysmal formation originating from the LCX and communicating with the RA. After aortic clamping, a right atriotomy was performed. The aneurysm was opened, then both communications (to the LCX and the RA) were closed using two autologous pericardial patches. A third patch was used to close the aneurysm after partial resection (Fig. 5).

**Figure 6:**
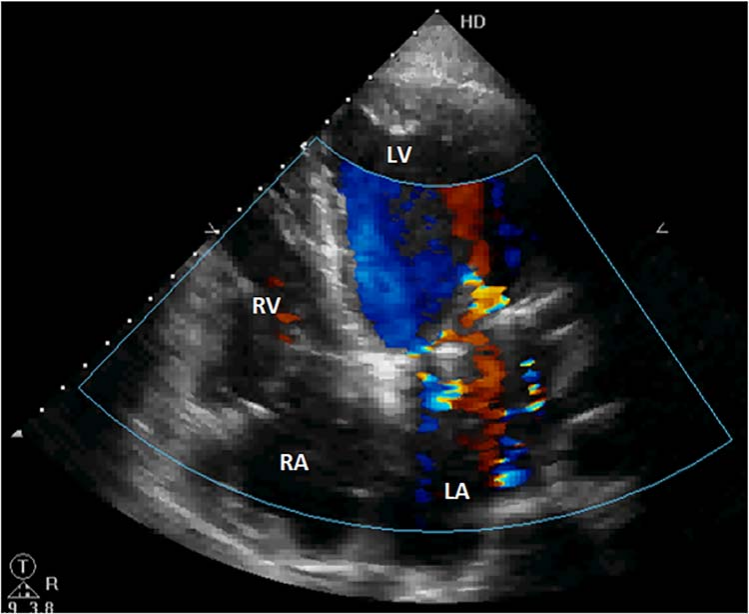
Postoperative TTE showing the disappearance of the right atrial shunt. RV, right ventricle; LV, left ventricle.

The patient was discharged after an uneventful postoperative course. The control TTE performed 2 weeks after the intervention showed that the right atrial shunt was gone (Fig. 6).

## DISCUSSION

CAF are exceptional, with an incidence of 0.002% in the general population, and account for 0.1% of coronary anomalies. The congenital etiology is by far the most common but acquired fistulas can develop after iatrogenic or traumatic events [1, 2]. The left anterior descending artery is the most frequent origin of CCF, with a prevalence of 42–66% in many recent registers. The LCX is the rarest origin, with a prevalence of 11–20% [3–5]. The site of drainage is also highly variable. It is generally accepted that most CCF drain in the right cavities and the pulmonary arteries [1]. The RA is a common site of drainage; Said [5] observed that half of CCF originating from the LCX shunt in the RA. Coronary aneurysms are common, with a prevalence of about 18% [2, 5]. This might be the result of a long-term left-to-right shunt, increasing the blood flow and leading to the destruction of the elastic fiber in the inner wall of the vessel. Ara et al. [6] found that the degree of fistula dilation is significantly correlated with mortality.

The majority of CAF are asymptomatic and are coincidentally found during angiographic procedures or cardiac surgery. Symptoms are present in 19–63% of patients, the most common being exertion dyspnea, which was the chief complaint of our patient. Larger CAF may cause congestive heart failure or angina because of a phenomenon of ‘myocardial steal’. Murmurs are a frequent physical finding; they may be systolic, diastolic or continuous [1, 2].

CA, despite being invasive, is the gold standard for the diagnosis of CAF. It has many limitations as it only shows an intraluminal image of the lesion and may be misleading when overlapping occurs between a tortuous fistula and adjacent cardiovascular structures. In the context of CAF, noninvasive imaging has seen a rapid rise for both diagnostic and follow-up purposes [7].

TTE with color Doppler is a first-line diagnostic tool. It provides a good view of the cardiac structures. Coronary artery dilation and turbulent flow because of the shunt can be observed. The main appeal of TTE is its rapidity and noninvasive nature, allowing for repeated controls, preoperatively and postoperatively. Unfortunately, it remains limited notably in patients with poor windows, and determining with certainty the origin of the CAF can be extremely difficult or impossible, as was the case in our report [1, 7].

The use of more advanced imaging techniques is key to the diagnosis of CAF. CCTA and CMR may play an important role in the diagnosis of these vascular anomalies. Both techniques are used to ascertain with precision the origin, course, size and site of drainage of a CAF as well as its relationship with adjacent structures. CMR can also be used to measure the blood flow within coronary arteries, and provide accurate estimations of cardiac output, shunt flow and valvular regurgitations. Late gadolinium enhancement sequences provide additional prognostic data about the myocardial wall status in case of ischemia. In case of aneurysmal CAF, cardiac CT is important to evaluate arterial wall thickness and morphology and detect atherosclerotic lesions or mural thrombi. Volume rendering technique reconstructions create an overview of the fistula and its course that proves extremely useful for surgical planning [7].

There are no guidelines on the management of CAF. Untreated larger fistulas may predispose to heart failure and myocardial ischemia; therefore, surgical treatment is often proposed to symptomatic patients and those with a large CAF. The surgical approach consists of closure of the fistula by ligation or patching of the orifice, sometimes alongside coronary artery bypass surgery. For smaller fistulas, a transcatheter approach using devices such as balloons, patches and plugs may be a more suitable alternative, as it has an excellent outcome with lesser morbidity and mortality, lower cost and shorter recovery time. The management of asymptomatic patients with small CAF remains controversial [8, 9].
